# Supplementary material for: An operationally implementable model for predicting the effects of an infectious disease on a comprehensive regional healthcare system
Source: PLoS One. 2021 Oct 20;16(10):e0258710. doi: 10.1371/journal.pone.0258710 (PMC8528335; doi:10.1371/journal.pone.0258710)
Supplement: Attachment — Submitted filename: RespReviewPLOS1.docx [file pone.0258710.s003.docx]

Dr. Robert Jeenchen Chen, MD, MPH

Academic Editor

PLOS ONE

August 11, 2021

Dear Dr. Chen,

Thank you for an expeditious review of our article entitled “An operationally implementable model for predicting the effects of an infectious disease on a comprehensive regional healthcare system” by Daniel Chertok, Chad Konchak, Nirav Shah, Kamaljit Singh, Loretta Au, Jared Hamernik, Brian Murray, Anthony Solomonides, Ernest Wang and Lakshmi Halasyamani. On behalf of all authors, I would like to thank the reviewers, Dr. Mahbub-Ul Alam and Dr. Ali Acar for their helpful comments that improved paper content and readability. In response to these comments, we made the following changes:

1. Page 1, Line 4 spells out “Illinois”, instead of “IL”, as the name of the US state;
2. Page 2, Lines 25 – 26 (previously, Line 24) spells out “Susceptible-Infected-Recovered” instead of “SIR”;
3. References have been reorganized to appear in the order of citation in the paper. We have added missing citations for the references specified by Reviewer #1. In addition, we verified that all references have a corresponding citation.
4. In the spirit of this comment and in order to improve readability, we converted all footnotes to inline comments, in compliance with the journal requirements. This change resulted in placing citations formerly appearing in the footnotes directly into the text of the paper.

In response to the comment from Reviewer #1 referencing Page 1, Line 3, we would like to point out that “NorthShore University HealthSystem” is the registered name of our organization, and this spelling needs to remain intact throughout the paper (please see [www.northshore.org](http://www.northshore.org) for reference).

Please note that, while the revised unmarked version of the manuscript contains citation links in proper order, the version containing tracked changes lists them in the order in which they appeared in the first submission. No references were removed or added to the revised version compared to the original.

Please do not hesitate to reach out to me with any remaining questions regarding our paper.

Sincerely,

Daniel Chertok, Ph.D.,

Senior Data Scientist – Clinical Analytics, Health Information Technology

NorthShore University HealthSystem,

4901 Searle Parkway, Suite 1450, Skokie, IL 60077, U.S.A.

Tel.: 847-982-5226	Email: dchertok@northshore.org
