# Supplementary material for: An operationally implementable model for predicting the effects of an infectious disease on a comprehensive regional healthcare system
Source: PLoS One. 2021 Oct 20;16(10):e0258710. doi: 10.1371/journal.pone.0258710 (PMC8528335; doi:10.1371/journal.pone.0258710)
Supplement: Attachment — Submitted filename: RespReviewPLOS1_2.docx [file pone.0258710.s004.docx]

Dr. Robert Jeenchen Chen, MD, MPH

Academic Editor

PLOS ONE

September 13, 2021

Dear Dr. Chen,

Thank you for a detailed review of our article entitled “An operationally implementable model for predicting the effects of an infectious disease on a comprehensive regional healthcare system” by Daniel Chertok, Chad Konchak, Nirav Shah, Kamaljit Singh, Loretta Au, Jared Hamernik, Brian Murray, Anthony Solomonides, Ernest Wang and Lakshmi Halasyamani. On behalf of all authors, I would like to thank anonymous reviewer #3 for helpful comments that improved paper content and readability. Below are our responses to these comments:

1. The choice of the SIR model was dictated primarily by the requirement of simplicity, tractability and distributability of the predictive software via an Excel spreadsheet. The use of a stochastic SIR model would have required making an assumption about the nature of the stochastic term and calibrating it to the observed infection dynamic. Likewise, IBM models introduce a level of complexity that require a substantial investment into their implementation and analysis. Dynamically adapting a model of this class to a rapidly changing situation on the ground would not be possible without more advanced computational tools and would deprive the user of the simplicity, transparency and flexibility of an Excel spreadsheet. Section “Materials and methods” of the paper has been expanded and references 5 – 9 added in order to address this concern directly in the paper.
2. No software, other than Microsoft Excel has been used in the preparation of the manuscript. Bootstrapping was performed using Excel Solver add-in called inside a VBA macro.
3. The exponential decay described in Fig. 1 stems from the fact that NorthShore University HealthSystem was at the forefront of initial testing and detection of COVID-19. For this reason, the number of positive cases during the early stages of the pandemic was disproportionately high compared with the (estimated) share of the corresponding geographical region. As the pandemic progressed, we observed a reduction in the aforementioned proportion to a “steady-state” level reflected in Eq. 18. Section “Estimating the number of potential NS patients” has been expanded to make this argument more transparent.
4. The exact count of true NorthShore patients at any given time depends of the definition of what constitutes a NS patient, moreover, many new patients presented at NS facilities through the emergency department without having had any previous contact with NorthShore. This necessitated the need to estimate the population in the catchment area that was likely to seek treatment at NS facilities. We are not aware of any sources that could help us estimate this number in an alternative way.
5. Weekends were implicitly addressed by averaging transmission coefficient over the preceding seven days. At one point in the pandemic, we attempted to extrapolate the transmission curve using a periodic term, however, those efforts did not lead to a reliable prediction. This argument was excluded from the paper for both brevity and clarity of exposition.
6. Subsection “Practical application” was added to “Results and Discussion” to include comments on the practical usefulness of the model.
7. The argument at the end of the conclusion was rephrased to be a more generic statement referencing the features, rather than the name, of COVID-19.

Please do not hesitate to reach out to me with any remaining questions regarding our paper.

Sincerely,

Daniel Chertok, Ph.D.,

Senior Data Scientist – Clinical Analytics, Health Information Technology

NorthShore University HealthSystem,

4901 Searle Parkway, Suite 1450, Skokie, IL 60077, U.S.A.

Tel.: 847-982-5226	Email: dchertok@northshore.org
